# Nano-Structural Characterization of Human Aponeurotic Tissue by Atomic Force Microscopy

**DOI:** 10.3390/biomedicines14020474

**Published:** 2026-02-21

**Authors:** Adelina Tanevski, Andreea Ludușanu, Bogdan Mihnea Ciuntu, Balan Gheorghe, Ștefan Octavian Georgescu, Valentin Bernic, Raoul-Vasile Lupușoru, Delia Gabriela Ciobanu Apostol, Ștefan Lucian Toma, Cristian Dumitru Lupașcu

**Affiliations:** 1 Faculty of Medicine, Grigore T. Popa University of Medicine and Pharmacy, 700115 Iasi, Romaniabogdan-mihnea.ciuntu@umfiasi.ro (B.M.C.); balan.gheorghe@umfiasi.com (B.G.);; 2 Faculty of Materials Science and Engineering, Gheorghe Asachi Technical University of Iași, 700050 Iași, Romania

**Keywords:** atomic force microscopy, aponeurotic tissue, nano-structural organization, surface topography, deflection contrast, collagen organization, extracellular matrix

## Abstract

**Background:** The structural integrity of the abdominal wall is critically dependent on the organization of aponeurotic tissue, a dense collagen-rich connective structure responsible for directional force transmission. While the clinical relevance of the aponeurosis is well recognized in abdominal wall reconstruction, its nano-scale structural organization remains insufficiently characterized. Atomic force microscopy (AFM) provides a suitable approach for investigating surface morphology and nano-architectural features of biological tissues. **Methods:** Human aponeurotic tissue samples were analyzed using atomic force microscopy operated in contact-mode deflection and topography imaging. Two-dimensional and three-dimensional surface topographies were acquired at the micrometer scale to assess nano-architectural organization. Areal surface roughness parameters (Sa, Sq, Sp, Sv, Sy) were calculated to quantify morphological heterogeneity. AFM deflection imaging was used to evaluate relative spatial variations in deflection imaging contrast under the applied scanning conditions across collagen-dense and interfibrillar regions. **Results:** AFM analysis revealed a well-organized fibrillar architecture with preferential orientation, consistent with the anisotropic organization of aponeurotic connective tissue. Deflection images demonstrated spatial heterogeneity in deflection contrast at the scanned scale, reflecting variations in the tip–sample interaction signal between collagen-dense and interfibrillar regions. Surface topography showed a continuous morphology with moderate height variations and smooth transitions between structural elements. Roughness parameters reflected a compact extracellular matrix organization within the scanned areas, without features suggestive of surface disruption. **Conclusions:** Atomic force microscopy enables detailed nano-scale structural characterization of human aponeurotic tissue and reveals spatial heterogeneity in deflection imaging contrast under specific contact-mode scanning conditions. These findings provide a baseline nano-scale descriptive reference dataset for macroscopically normal aponeurotic tissue, supporting future comparative investigations without implying validated mechanical differences or direct tissue–implant interaction analysis within the present study.

## 1. Introduction

Abdominal wall integrity is clinically central to hernia repair and abdominal wall reconstruction, where both prosthetic reinforcement and optimized closure strategies are employed to reduce failure and recurrence rates [1,2]. Biomechanical investigations have consistently highlighted the importance of native abdominal wall tissue properties in determining surgical outcomes. Within this complex anatomical system, the aponeurosis represents a dense, collagen-rich connective structure responsible for the transmission and redistribution of mechanical forces generated by the abdominal musculature. Its functional integrity is essential for maintaining abdominal wall stability, and its impairment plays a key role in the pathogenesis of primary and incisional hernias, as well as in postoperative failure following abdominal wall reconstruction [3,4,5].

Over recent decades, the widespread adoption of tension-free repair techniques using synthetic meshes has led to a substantial reduction in recurrence rates in abdominal wall hernia surgery [6,7]. However, these advances have simultaneously brought attention to a new spectrum of clinical challenges, including seroma formation, chronic pain, impaired abdominal wall compliance, and mesh-related complications [3,8]. Increasing evidence indicates that such outcomes are influenced not only by surgical technique or prosthetic material selection, but also by the intrinsic biological and structural characteristics of the host tissue interacting with the implant [9,10]. In this context, the aponeurosis constitutes a critical, yet still insufficiently characterized, interface between native tissue and synthetic materials. From a biological standpoint, aponeurotic tissue is composed predominantly of highly organized collagen fibers embedded within an extracellular matrix that confers both tensile strength and controlled deformability [11,12]. This organization is inherently anisotropic, reflecting the directional mechanical demands imposed on the abdominal wall [13,14]. While macroscopic biomechanical testing has yielded valuable information regarding the tensile behavior of aponeurotic structures, such approaches are inherently limited in their ability to capture local heterogeneities in collagen organization, surface architecture, and interaction behavior that may be highly relevant for tissue–implant integration and long-term functional adaptation [5,15].

At the microscale, conventional optical microscopy provides valuable insight into overall tissue morphology and collagen fiber arrangement, offering essential structural context for higher-resolution investigations. Although optical techniques do not resolve nanoscale surface features or local interaction behavior, they allow visualization of global architectural patterns, fiber orientation, and structural heterogeneity that may influence nanoscale measurements. Integrating optical microscopy with AFM-based analysis therefore supports a multi-scale characterization approach, facilitating the correlation between microscale collagen architecture and nanoscale topographical and deflection-related features.

Atomic force microscopy (AFM) has emerged as a powerful tool for investigating biological tissues at the micro- and nano-scale [16,17]. Unlike conventional imaging techniques, AFM allows simultaneous assessment of surface morphology and relative local mechanical response with high spatial resolution [18]. Previous AFM studies of soft tissues have demonstrated that variations in collagen organization, fibrillar density, and extracellular matrix composition can markedly influence local mechanical behavior, even within macroscopically homogeneous samples [15,19]. Despite its clear clinical relevance, however, the nano-scale architecture and local mechanical heterogeneity of human aponeurotic tissue remain poorly documented in the literature. A detailed AFM-based characterization of aponeurotic tissue is therefore particularly relevant in the context of abdominal wall reconstruction, where synthetic meshes are implanted in direct contact with native connective tissue [15]. Establishing the baseline nano-structural organization of macroscopically intact human aponeurosis has been discussed in the literature as a relevant step toward future comparative analyses involving prosthetic materials and post-implant tissue responses [16,20]. Such reference data may serve as a descriptive baseline for future studies, without implying direct interpretation of tissue–mesh interactions, integration behavior, or clinical outcomes.

The aim of the present study is to perform a comprehensive nano-structural characterization of human aponeurotic tissue using atomic force microscopy. By combining contact-mode AFM deflection imaging with two-dimensional and three-dimensional surface topography analysis, this work focuses on documenting collagen fiber organization, surface morphology, and spatial heterogeneity in deflection and interaction contrast at the nano-scale. The findings are intended to establish baseline reference dataset for future comparative investigations, without drawing comparative or causal conclusions within the present study.

Despite its clinical relevance, the nano-scale literature on human aponeurotic tissue remains limited and fragmented, with few AFM-based studies providing systematic quantitative descriptions of surface roughness parameters, anisotropic fibrillar organization, and spatial heterogeneity.

## 2. Materials and Methods

### 2.1. AFM Instrumentation and Software

Atomic force microscopy (AFM) investigations were performed using an EasyScan 2 atomic force microscope (Nanosurf AG, Liestal, Switzerland). Image acquisition and data processing were carried out using Nanosurf EasyScan 2 image analysis software (Nanosurf AG, Liestal, Switzerland).

### 2.2. Tissue Samples

A total of seven human aponeurotic tissue samples were analyzed. Specimens were harvested intraoperatively from the linea alba of middle-aged adult patients (four males and three females) undergoing scheduled abdominal surgery for benign non-abdominal-wall pathology, including colorectal and gynecological conditions. All patients had body mass index values ranging between 18.5 and 21 kg/m^2^ and presented an intact abdominal wall, with no clinical or intraoperative evidence of abdominal wall defects, previous surgical remodeling of the abdominal wall, or local inflammatory changes at the sampling site. All samples were obtained from macroscopically normal linea alba tissue. Tissue specimens were collected from the midline linea alba, at a standardized anatomical location, and consisted exclusively of aponeurotic tissue, with careful avoidance of adjacent muscular layers. As the linea alba represents a midline structure, no left–right side differentiation was applicable.

**Figure 1:**
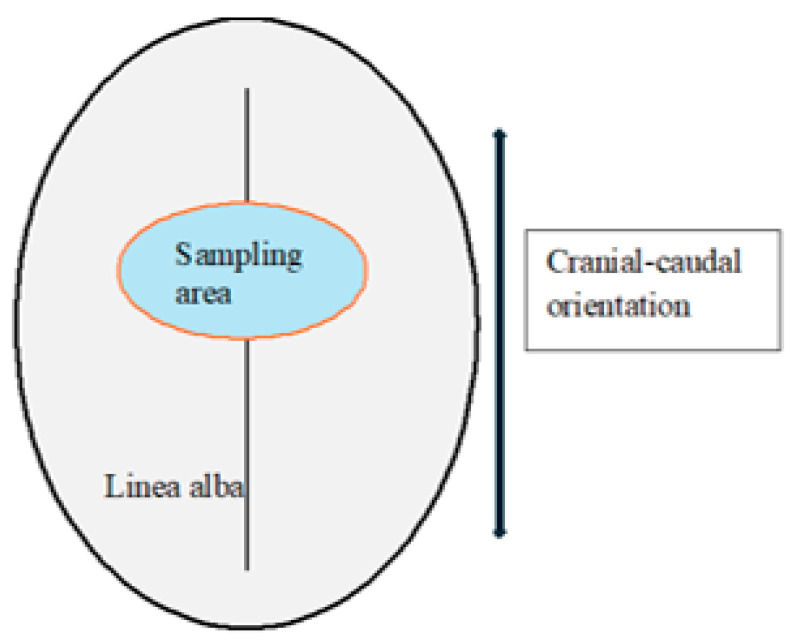
Schematic representation of the standardized sampling location and orientation of aponeurotic tissue along the midline linea alba. The diagram is intended for illustrative purposes only.

Specimen orientation was preserved during excision and preparation, with the longitudinal axis of each sample aligned parallel to the predominant collagen fiber direction of the linea alba. A schematic representation of the standardized sampling location and orientation is provided in Figure 1.

Immediately after excision, each aponeurotic tissue specimen was divided into two portions to allow parallel analyses. One portion was processed for conventional optical microscopy, while the second portion was prepared for atomic force microscopy. Tissue fragments intended for AFM analysis (sub-centimeter in size) were immersed in RNAlater^®^ solution (Thermo Fisher Scientific, Waltham, MA, USA) to preserve tissue integrity and minimize post-excision degradation. Prior to AFM preparation, samples were briefly rinsed with DPBS (1×, pH 7.0) (Gibco™, Thermo Fisher Scientific, Waltham, MA, USA) to remove residual storage solution.

### 2.3. Optical Microscopy

The human aponeurotic tissue samples were processed for conventional optical microscopy in order to obtain complementary morphological information and to provide microscale structural context for the AFM analysis. Tissue fragments were fixed in buffered formalin, routinely processed, embedded in paraffin, and sectioned at standard thickness. The resulting sections were stained using hematoxylin and eosin (H&E) according to standard histological protocols.

Optical microscopy was performed using a light microscope equipped with digital image acquisition. Representative images were captured at low, medium, and high magnifications (×4, ×10, and ×20) to evaluate overall tissue architecture, collagen fiber organization, cellular composition, and vascular features. Particular attention was paid to collagen bundle orientation, fiber parallelism, and the presence and distribution of resident connective tissue cells, including fibrocytes and fibroblasts.

Optical microscopy was employed as a qualitative, complementary technique to support the interpretation of AFM findings. The obtained micrographs were not subjected to quantitative histomorphometric analysis, but were used to facilitate the correlation between microscale structural organization and nanoscale surface topography and deflection-related features observed by atomic force microscopy.

### 2.4. Sample Preparation for AFM Analysis

To ensure stable tissue immobilization and reproducible tip–sample contact during scanning, aponeurotic tissue samples were fixed onto commercially available poly-L-lysine (PLL)-coated glass microscope slides (Bio-Optica Milano S.p.A., Milan, Italy), which promote electrostatic adhesion of biological specimens. Prior to tissue placement, the PLL-coated slides were equilibrated at room temperature. Small aponeurotic tissue fragments (approximately 3.0 × 3.0 × 1.0 mm) were placed onto the treated glass surface and gently pressed to ensure adequate contact without inducing mechanical deformation. The tissue–slide assemblies were incubated for 20–30 min at room temperature to allow stable electrostatic attachment.

Following immobilization, samples were transferred to phosphate-buffered saline (DPBS 1×, pH 7.0) for rinsing and short-term hydration. For AFM measurements conducted in air, samples were mounted immediately after removal from DPBS and imaging was initiated without delay to minimize dehydration-related artifacts. All AFM measurements were performed within 24 h of tissue harvesting to minimize post-excision alterations of surface morphology. This preparation method provided sufficient mechanical stability for contact-mode AFM scanning while preserving the native nano-structural features of the aponeurotic surface. Immediately after excision, tissue samples were temporarily preserved in RNAlater to limit post-excision degradation, rinsed in DPBS prior to imaging, and mounted for AFM analysis in air within 8 h. This procedure was selected to preserve structural integrity rather than native mechanical properties. Atomic force microscopy was consistently performed on the same anatomical surface across all samples, corresponding to the exposed aponeurotic plane obtained during specimen preparation.

### 2.5. AFM Probes

AFM imaging was performed using PPP–NCSTAuD probes (Nanosensors, Neuchâtel, Switzerland). The probes were gold-coated on the detector-facing side to enhance signal sensitivity. According to the manufacturer’s specifications, these highly doped silicon cantilevers have a length of 150 μm and a nominal spring constant of 0.4 N/m, with a typical tip radius <7 nm. AFM imaging was performed in contact mode over scan areas of 2 × 2 µm at a scan rate of 0.5–0.8 Hz. The contact force (setpoint) was maintained at the minimum value required to ensure stable tip–sample interaction, and feedback gains were adjusted to minimize lateral forces and avoid oscillatory responses during scanning. AFM deflection images were interpreted exclusively in a qualitative and comparative manner, as indicators of relative spatial variations in the tip–sample interaction signal. No absolute or quantitative nanomechanical parameters were derived from deflection data. AFM imaging was performed over scan areas of 2 × 2 µm at a scan rate of 0.5–0.8 Hz. The contact force (setpoint) was maintained at the minimum value required for stable tip–sample interaction, and feedback gains were adjusted to minimize lateral forces during scanning.

### 2.6. Imaging Conditions

AFM measurements were conducted in air at room temperature. Surface imaging was performed in contact mode, ensuring continuous tip–sample interaction during scanning. Height and deflection channels were recorded simultaneously for all measurements. Localized AFM scans were acquired over micrometer-scale areas of 2 × 2 µm. Prior to quantitative analysis, AFM height images were plane-leveled and corrected line-by-line to remove global tilt and scanner-related artifacts; no smoothing or filtering procedures were applied. 

### 2.7. Deflection Imaging

AFM deflection imaging was used to assess relative spatial variations in the local tip–sample interaction during contact-mode scanning. In this mode, the deflection signal reflects cantilever bending induced by mechanical interaction with the tissue surface. Deflection contrast was interpreted qualitatively, in a comparative manner within and between scans, as an indicator of local heterogeneity in surface interaction properties. No absolute mechanical parameters were derived from deflection images.

### 2.8. Surface Topography and Roughness Analysis

Two-dimensional height images were acquired simultaneously with deflection data to characterize surface morphology and nano-architectural organization. Three-dimensional surface reconstructions were generated from height maps to visualize the spatial distribution of surface relief. Standard surface roughness parameters were calculated, including the arithmetic mean height (Sa), the root mean square roughness (Sq), the maximum peak height (Sp), the maximum valley depth (Sv), and the total height (Sy). These parameters were used to describe surface continuity, height distribution, and morphological heterogeneity.

### 2.9. Surface Profile Extraction

Surface profiles were extracted from selected regions of interest within the height maps to visualize local height variations and to correlate topographic depressions and elevations with structural features observed in AFM images. Profile analysis was performed along representative scan lines within the imaged areas.

### 2.10. Data Analysis

AFM data were analyzed descriptively. The analysis focused on the characterization of fibrillar organization, preferential structural orientation, surface continuity, spatial heterogeneity in deflection contrast, and nano-scale roughness features. No absolute mechanical parameters were calculated, and no inferential statistical analysis was performed. For each patient, three independent AFM regions of interest (ROI) of 2 × 2 µm were analyzed, selected from macroscopically intact areas of the aponeurotic tissue. Roughness parameters were calculated for each ROI, and patient-level values were obtained as the mean of the three scans. Group data are reported descriptively as mean ± standard deviation (*n* = 7), without inferential statistical analysis.

## 3. Results

### 3.1. Optical Microscopy Findings

**Figure 2:**
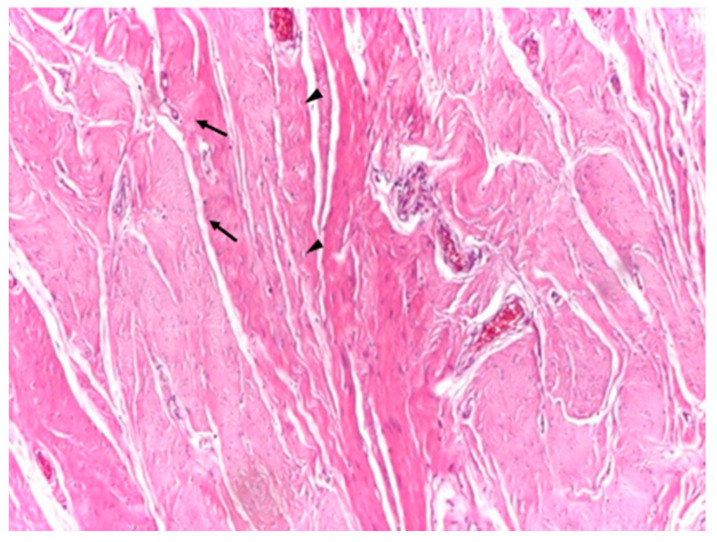
Representative low-magnification optical micrograph of human aponeurotic tissue highlighting parallel and orderly arranged collagen fiber bundles. Arrows indicate fibroblasts with rounder nuclei, while arrowheads indicate fibroblasts with elongated fusiform nuclei in longitudinal section. *Hematoxylin and eosin staining (H&E); original magnification ×4*.

Optical microscopy of human aponeurotic tissue revealed a well-preserved connective architecture characterized by dense, predominantly parallel collagen fiber bundles forming an organized structural framework. At low magnification (×4), the aponeurotic fragments displayed a compact and continuous collagenous matrix with a clear preferential fiber orientation, consistent with the load-bearing function of the abdominal wall aponeurosis (Figure 2).

**Figure 3:**
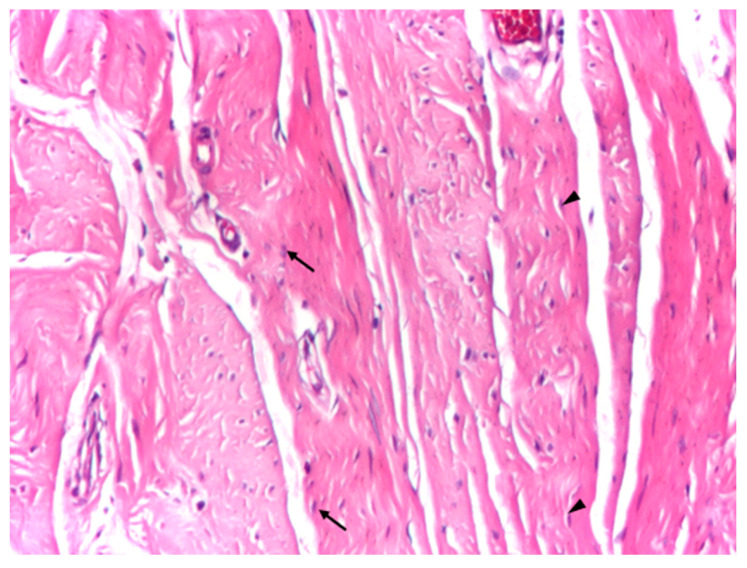
Optical microscopy of human aponeurotic tissue showing predominantly fibrocytes with sparse elongated fibroblasts within an organized collagenous matrix. Arrows indicate fibroblasts with rounder nuclei, while arrowheads indicate fibroblasts with elongated fusiform nuclei in longitudinal section. *Hematoxylin and eosin staining (H&E); original magnification ×10*.

At intermediate magnification (×10), the aponeurotic tissue exhibited a sparse cellular component embedded within the collagen matrix, consisting predominantly of fibrocytes with occasional elongated fibroblasts. The collagen bundles maintained a uniform and parallel arrangement, without evidence of structural disruption or pathological remodeling (Figure 3).

**Figure 4:**
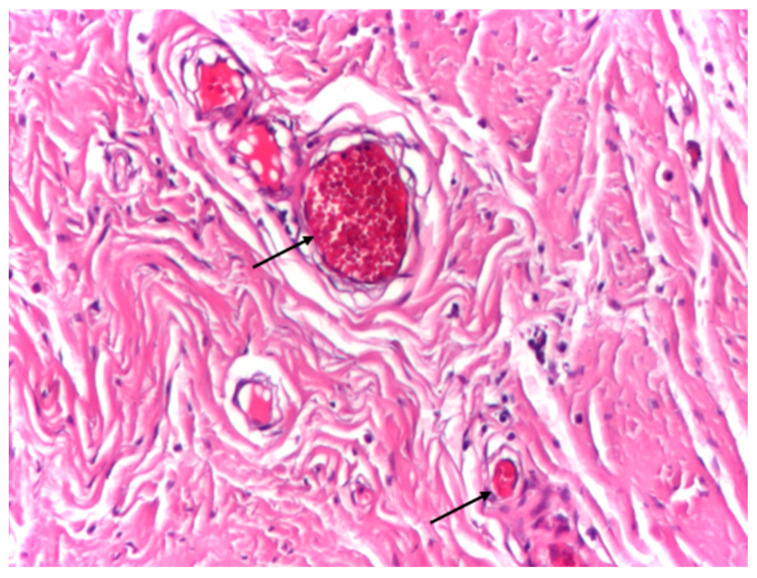
Optical microscopy of human aponeurotic tissue showing vascular structures with preserved wall integrity and discrete intraluminal stasis, without associated inflammatory reaction. Arrows indicate small vascular structures with visible lumina containing erythrocytes. *Hematoxylin and eosin staining (H&E); original magnification ×10*.

Vascular structures were infrequently observed within the aponeurotic tissue and displayed preserved wall integrity, with discrete intraluminal stasis. No perivascular inflammatory infiltrate, endothelial damage, or pathological vascular remodeling was identified in the surrounding connective tissue (Figure 4).

**Figure 5:**
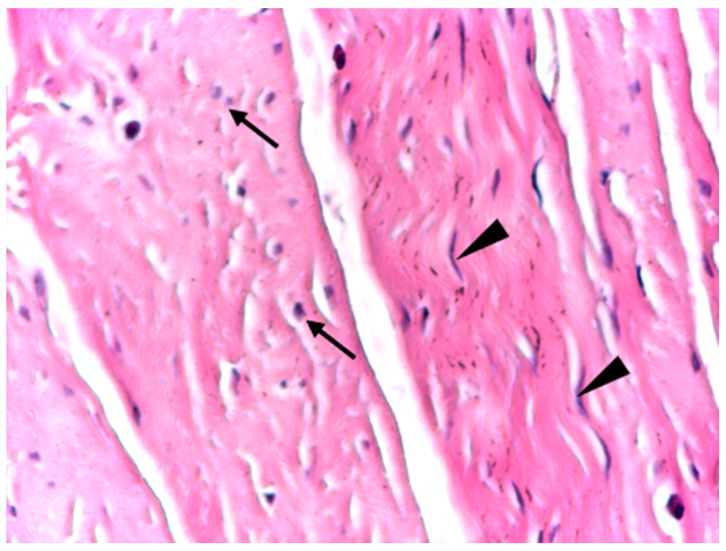
High-magnification optical micrograph of human aponeurotic tissue showing fusiform active fibroblasts alongside fibrocytes within the collagenous matrix. Arrows indicate fibroblasts with rounder nuclei in cross section, while arrowheads indicate fibroblasts with elongated fusiform nuclei in longitudinal section. *Hematoxylin and eosin staining (H&E); original magnification ×20*.

At high magnification (×20), optical microscopy highlighted physiological cellular heterogeneity within the aponeurotic connective tissue, characterized by the presence of fusiform, metabolically active fibroblasts alongside fibrocytes. These cells were distributed between collagen bundles without disrupting the overall structural continuity of the extracellular matrix or inducing local inflammatory changes (Figure 5).

**Figure 6:**
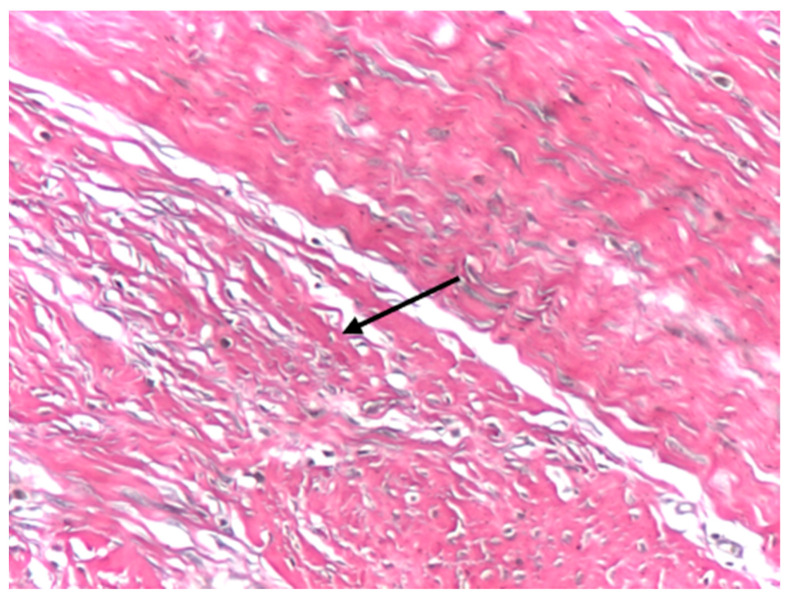
High-magnification optical micrograph of human aponeurotic tissue. Arrows indicate dense, wavy collagen fiber bundles highlighted in longitudinal section, by Van Gieson’s trichrome staining. *Van Gieson’s trichrome staining; original magnification ×10*.

Van Gieson’s trichrome staining was used as a collagen-specific histological method to complement the hematoxylin–eosin findings. At ×10 magnification, aponeurotic tissue exhibited dense, wavy, and well-organized collagen fibers arranged in parallel bundles, with fibrocytes and occasional fibroblasts interspersed between collagen fascicles (Figure 6). The overall architecture appeared continuous and preserved, consistent with macroscopically normal aponeurotic connective tissue. The histological assessment was qualitative and intended to support the structural context of the AFM analysis.

### 3.2. AFM Deflection Imaging of Aponeurotic Tissue

**Figure 7:**
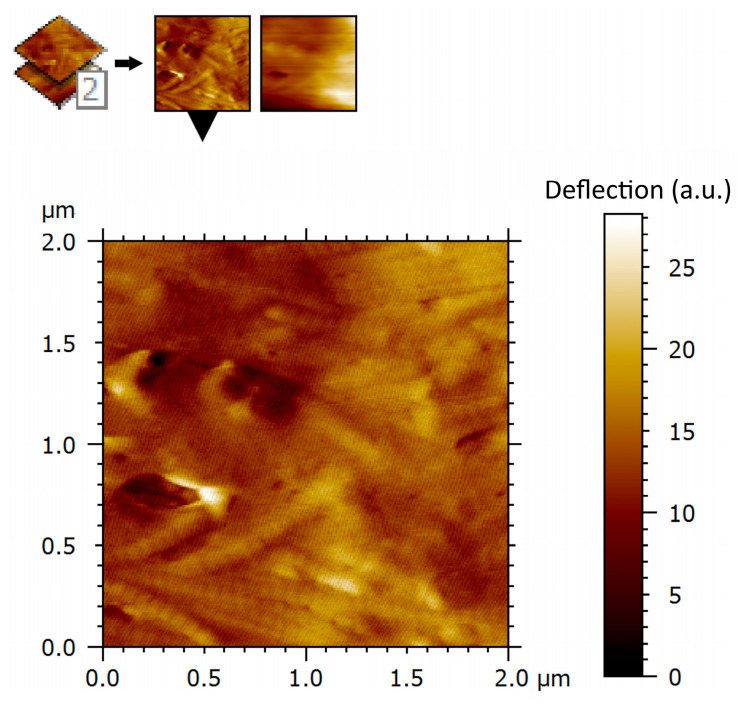
AFM deflection contrast of human aponeurotic tissue at the nanoscale. Deflection contrast is shown in arbitrary units (a.u.) and reflects relative variations in the tip–sample interaction signal. AFM imaging parameters: contact mode; scan area: 2 × 2 µm; scan rate: 0.5–0.8 Hz; minimal contact force (setpoint); feedback gains optimized to minimize lateral forces.

Atomic force microscopy deflection imaging revealed a well-organized fibrillar architecture of the aponeurotic tissue surface. At the scanned scale of 2 × 2 µm, elongated structures with a preferential orientation were consistently observed, forming an anisotropic surface pattern. The partial parallel alignment of these structures indicates a non-amorphous, anisotropic organization. The deflection contrast reveals marked spatial heterogeneity in the tip–sample interaction signal across the aponeurotic surface. Brighter regions correspond to areas with increased deflection contrast and spatially overlap with collagen-dense structures, whereas darker regions are associated with interfibrillar areas. These differences indicate nano-scale heterogeneity in the interaction signal across the scanned surface, which correlates with the local fibrillar organization observed in the corresponding AFM topography (Figure 7). This observation is limited to a qualitative and comparative description of imaging contrast and does not imply quantified differences in mechanical or physiological properties of the tissue.

### 3.3. Two-Dimensional Surface Topography

**Figure 8:**
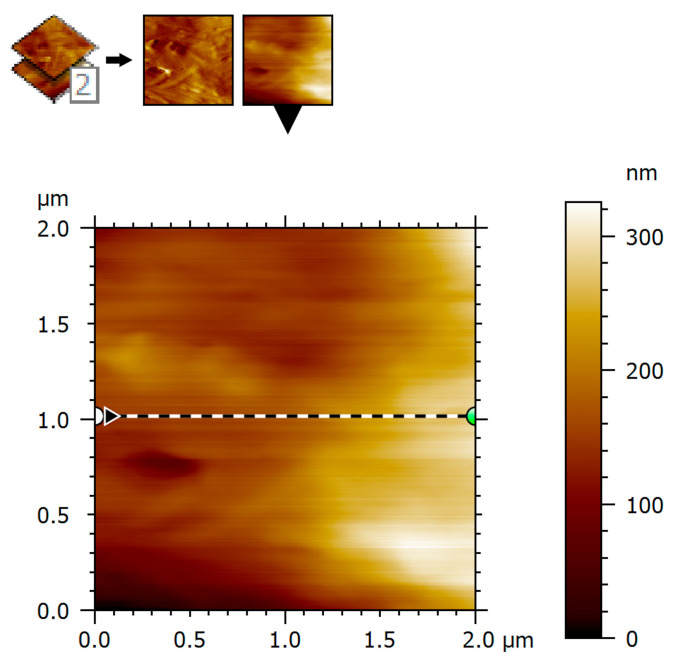
Two-dimensional AFM surface topography of human aponeurotic tissue. Surface height is displayed in nanometers (nm). AFM imaging parameters: contact mode; scan area: 2 × 2 µm; scan rate: 0.5–0.8 Hz; minimal contact force (setpoint); feedback gains optimized to minimize lateral forces.

Two-dimensional AFM topography demonstrated a continuous surface morphology with moderate height variations and smooth transitions between surface features. Within the scanned regions, no abrupt discontinuities, sharp edges, or well-defined pores were observed, supporting preservation of the imaged tissue architecture. The surface exhibited elongated, gently undulating structures oriented preferentially along a dominant direction. Gradual transitions between ridges and valleys were evident and corresponded to collagen fiber fascicles embedded within the extracellular matrix. This topographic organization is consistent with the anisotropic structural arrangement of aponeurotic tissue and its role in directional force transmission (Figure 8).

It should be emphasized that these observations are restricted to the scanned 2 × 2 µm areas and do not exclude the presence of pores or structural features at larger length scales.

### 3.4. Three-Dimensional AFM Topography

**Figure 9:**
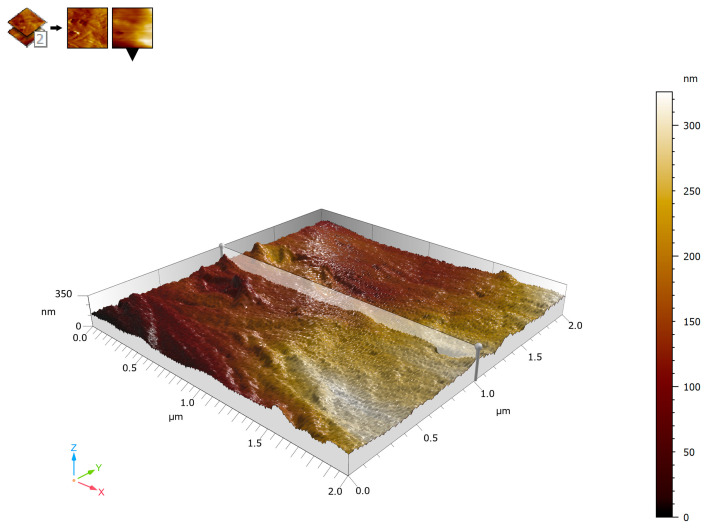
Three-dimensional AFM surface topography of human aponeurotic tissue. Surface height is displayed in nanometers (nm). AFM imaging parameters: contact mode; scan area: 2 × 2 µm; scan rate: 0.5–0.8 Hz; minimal contact force (setpoint); feedback gains optimized to minimize lateral forces.

Three-dimensional reconstruction of the AFM topography provided enhanced visualization of the spatial distribution of surface relief. The surface appeared continuous and compact, with an undulating morphology and gradual height variations. The Z-range after line fitting was approximately ±60–70 nm, indicating a moderate surface relief compatible with a dense extracellular matrix rather than a granular or degraded surface. The three-dimensional representation emphasized the preferential orientation of elongated ridges and valleys corresponding to anisotropically arranged collagen fiber bundles. This anisotropy was more clearly evident in the three-dimensional projection than in the two-dimensional images, reinforcing the structural specialization of the aponeurosis for directional mechanical load transmission (Figure 9).

### 3.5. Surface Roughness Parameters

A descriptive analysis of surface roughness parameters (Table 1), performed at the patient level (*n* = 7) using the mean values of three independent AFM scan areas per sample, revealed moderate and consistent Sa, Sq, Sp, Sv, and Sy values, summarized in Table 2.

Quantitative roughness analysis revealed moderate values of the arithmetic mean height (Sa), consistent with a compact and structurally intact extracellular matrix. The values reported in Table 2 are derived from representative 2 × 2 µm AFM scan areas and reflect the overall organization of collagen fibers rather than the presence of pronounced surface asperities or morphological degradation. Similar roughness characteristics were observed across all analyzed samples.

For each patient, roughness parameters were calculated as the mean of three independent AFM regions of interest (ROI) of 2 × 2 µm. In total, 21 AFM scans were analyzed (7 patients × 3 ROI). Group values are reported as mean ± standard deviation (*n* = 7).

The root mean square roughness (Sq) exceeded Sa, indicating a non-uniform distribution of surface heights with localized variations. This relationship suggests spatial heterogeneity related to differences in fibrillar density and interfibrillar spacing, without evidence of chaotic or irregular surface features. Comparable surface patterns were consistently identified across the scanned regions of all seven samples. The ratio between maximum peak height and maximum valley depth (Sp/|Sv|) was close to unity, indicating that surface peaks and valleys were of comparable magnitude and lacked isolated extreme features. The total height parameter (Sy) reached medium values of approximately 143 nm, indicating a moderate height difference between surface peaks and valleys and showing no features suggestive of fissures, pores, or structural ruptures within the analyzed areas.

### 3.6. Surface Profile Analysis

**Figure 10:**
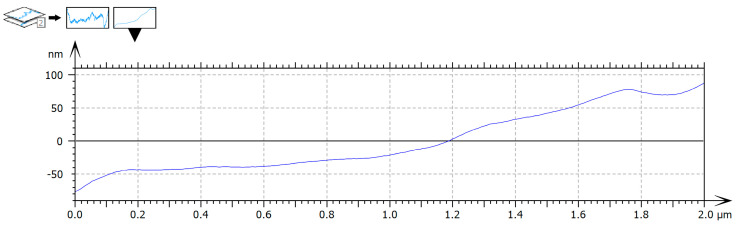
AFM surface height profile extracted from the topography of human aponeurotic tissue. Height variations are displayed in nanometers (nm) along a representative line profile. AFM imaging parameters: contact mode; scan area: 2 × 2 µm; scan rate: 0.5–0.8 Hz; minimal contact force (setpoint); feedback gains optimized to minimize lateral forces.

Surface profile analysis extracted from representative regions revealed a central depression flanked by elevated areas. The central region exhibited a lower height profile, while the lateral elevations spatially overlapped with collagen-rich structures displaying increased topographic prominence. The surface profile findings were consistent with the spatial heterogeneity observed in both deflection imaging and topographic maps, illustrating variations in surface organization across the aponeurotic tissue. Height variations occurred smoothly along the profile, without abrupt transitions, further supporting the integrity and organized architecture of the tissue (Figure 10).

## 4. Discussion

### 4.1. Nano-Scale Architecture of Normal Aponeurotic Tissue

The present study provides a detailed nano-scale characterization of human aponeurotic tissue using atomic force microscopy, with a focus on surface morphology, fibrillar organization, and spatial heterogeneity in deflection contrast during contact-mode imaging. By combining deflection imaging with two-dimensional and three-dimensional topographic analysis, the findings offer a high-resolution descriptive overview of the aponeurotic surface architecture without relying on absolute mechanical measurements.

AFM imaging revealed elongated, partially parallel surface structures with a preferential orientation, consistent with the collagen-dominated composition of aponeurotic connective tissue. Such anisotropic organization represents a known structural characteristic of dense collagen-rich tissues and has been previously described using histological and biomechanical approaches [21,22,23]. In the present study, this organization is observed and described at the nano-scale, supporting the preservation of collagen bundle orientation within the scanned regions rather than constituting a novel functional finding.

### 4.2. Spatial Heterogeneity and Deflection Contrast

It is important to emphasize that the heterogeneity observed in AFM deflection images reflects relative variations in imaging contrast associated with local fibrillar organization, rather than quantified differences in mechanical or physiological properties. In the absence of force–indentation measurements or modulus mapping, the interpretation is intentionally limited to a structural and comparative level. Within this framework, regions of increased deflection contrast spatially overlapping with collagen-dense bundles and lower-intensity regions corresponding to interfibrillar areas are consistent with patterns previously reported in AFM studies of collagen-rich biological tissues, where deflection contrast variations are associated with differences in fibrillar density, orientation, and packing [21,24]. The heterogeneity described here therefore reflects the intrinsic structural complexity of normal aponeurotic tissue, rather than pathological alteration.

Two-dimensional AFM topography further supports this interpretation by demonstrating a continuous and compact surface morphology with moderate height variations and smooth transitions between ridges and valleys. Such surface continuity is consistent with intact collagen-dominated connective tissues and contrasts with the disrupted or irregular topographies reported in altered tissue states or certain mesh–tissue interaction contexts [4,15,18]. In the present study, these topographic features are interpreted as descriptors of preserved nano-structural organization at the scanned micrometer scale.

### 4.3. Three-Dimensional Organization and Surface Roughness

Three-dimensional surface reconstruction enhanced visualization of the spatial arrangement of topographic features and further highlighted the anisotropic organization of ridges and valleys. The moderate Z-range observed is consistent with a compact extracellular matrix rather than a granular or degraded structure. Previous AFM investigations have demonstrated that three-dimensional visualization is particularly valuable for identifying directional organization and spatial heterogeneity in fibrous tissues, which may be underestimated in two-dimensional projections alone [25,26,27]. In the case of aponeurotic tissue, this anisotropic organization is consistent with the known structural arrangement of collagen fibers in dense connective tissues.

Quantitative roughness analysis supported the interpretation of a structurally intact and organized surface. The Sa and Sq values measured in the present study (on the order of tens of nanometers), together with a total height (Sy) of approximately 140 nm, are comparable in magnitude to roughness values reported in AFM studies of collagen-rich connective tissues such as fascia and tendon, despite differences in tissue type, scan size, and imaging conditions. Moderate arithmetic mean height (Sa) values, together with higher root mean square roughness (Sq), indicate a non-uniform height distribution with localized variations related to differences in fibrillar density and interfibrillar spacing. Similar roughness profiles have been reported for collagen-based biological surfaces and are considered characteristic of functional fibrous tissues rather than disorganized or pathological matrices [21,26,28]. The Sp/Sv ratio close to unity and the moderate total height (Sy) value indicate the absence of isolated peaks, deep valleys, fissures, or pores, reinforcing the interpretation of a uniform and biologically coherent surface architecture.

Surface profile analysis further revealed smooth height variations with gradual transitions between elevated and depressed regions, corresponding to differences in surface organization observed in the topographic maps. Such profiles are consistent with the composite nature of aponeurotic tissue, in which collagen fibers and extracellular matrix components interact to generate a continuous yet heterogeneous structural arrangement [29,30,31]. Comparable profile characteristics have been described in AFM studies of fascia and tendon [32].

### 4.4. Correlation with Microscale Morphology

The nano-scale AFM findings are consistent with the preserved microscale architecture observed by optical microscopy in the present study, which demonstrated parallel collagen fiber bundles, sparse cellularity, and the absence of inflammatory or degenerative changes. Together, these observations support the interpretation that the AFM-derived heterogeneity reflects inherent structural organization rather than processing-related artifacts or pathological remodeling. The integration of optical microscopy and AFM therefore enables a multi-scale structural framework, linking collagen architecture at the microscale with surface morphology and interaction heterogeneity at the nano-scale.

### 4.5. Clinical Relevance and Implications for Abdominal Wall Reconstruction

From a clinical perspective, defining a nano-scale reference profile for macroscopically normal aponeurotic tissue is highly relevant for abdominal wall reconstruction. Synthetic meshes are implanted in direct contact with the aponeurosis, and their integration depends not only on bulk material properties but also on surface topography and local structural compatibility. Variations in tissue–implant interactions have been associated with excessive fibrosis, increased stiffness, chronic pain, and impaired functional outcomes [3]. The present findings provide a biological benchmark against which the nano-structural characteristics of prosthetic materials can be compared in future investigations.

### 4.6. Limitations and Future Directions

Although contact-mode AFM imaging in air may induce tip-related deformation or dehydration artifacts in ultrasoft biological tissues, the present study focused on normal human aponeurotic tissue, a dense collagen-rich structure with comparatively higher mechanical stability. Under the conservative scanning conditions employed, no evidence of surface damage, lateral dragging, or plastic deformation was observed. Repeated scans performed over the same regions under identical conditions did not reveal detectable changes in surface morphology, indicating that the imaging protocol did not induce measurable surface modification within the resolution limits of the instrument.

Several limitations should nevertheless be acknowledged. The present analysis was intentionally descriptive and did not include absolute mechanical measurements, viscoelastic modeling, or inferential statistical analysis of inter-individual variability. In addition, surface characterization was limited to micrometer-scale AFM scan areas, which may not capture all aspects of larger-scale tissue heterogeneity, including features such as micro-porosity.

As a next step, a direct comparative experimental design should include AFM-based surface topography and roughness analysis of clinically used prosthetic mesh materials alongside normal human aponeurotic tissue, performed under identical scanning conditions. This approach should be complemented by quantitative nanomechanical mapping in liquid environments (e.g., force–indentation or PeakForce QNM) to assess elastic modulus and adhesion across both tissue and mesh surfaces. Such a framework would enable systematic comparison of collagen-dominated tissue architecture and mesh surface topography at the same length scale, providing a rational basis for future tissue–mesh interaction studies.

## 5. Conclusions

This study demonstrates that atomic force microscopy provides detailed nano-scale insight into the surface architecture of human aponeurotic tissue. By combining deflection imaging with two-dimensional and three-dimensional topographic analysis, a descriptive characterization of fibrillar organization, preferential orientation, and moderate surface roughness was obtained, consistent with the collagen-dominated composition of macroscopically normal aponeurosis.

The findings reveal pronounced local heterogeneity in the deflection contrast across the tissue surface, correlated with differences in fibrillar density and interfibrillar organization. Within the scope of the present study, this heterogeneity is interpreted as a structural feature of normal aponeurotic tissue, rather than as evidence of pathological alteration.

Quantitative roughness parameters and surface profile analysis further support the presence of a continuous, non-porous, and structurally intact surface, without features suggestive of degradation or architectural disruption. The observed anisotropic nano-structural organization reflects the known directional arrangement of collagen fibers within aponeurotic tissue, as described for dense collagen-rich connective tissues.

Collectively, these results establish a nano-scale reference profile for macroscopically normal human aponeurotic tissue. This reference may serve as a baseline for future comparative investigations involving prosthetic materials or pathological tissue, without implying direct analysis of tissue–implant interactions or clinical outcomes within the present study.

## Figures and Tables

**Table 1:** AFM surface roughness parameters.

Parameter	Description	Unit
Area	Analyzed surface area	µm^2^
Sa	Arithmetic mean height	nm
Sq	Root mean square roughness	nm
Sy	Total height (peak-to-valley)	nm
Sp	Maximum peak height	nm
Sv	Maximum valley depth	nm

**Table 2:** AFM surface roughness parameters of human aponeurotic tissue.

Patient	Sa (nm)	Sq (nm)	Sy (nm)	Sp (nm)	Sv (nm)	ROI/Patients
P1	17.34	22.84	140.75	76.12	−60.15	3
P2	18.04	20.42	139.26	77.38	−68.92	3
P3	16.36	18.96	138.84	77.81	−64.98	3
P4	18.53	23.18	146.46	76.48	−67.86	3
P5	16.46	22.18	148.14	75.69	−66.44	3
P6	18.44	21.03	141.26	76.18	−65.78	3
P7	16.76	22.76	144.63	78.31	−66.87	3
Media ± SD	17.42 ± 0.93	21.62 ± 1.55	142.76 ± 3.65	76.85 ± 0.98	−65.86 ± 2.83	

## Data Availability

The datasets generated and analyzed during the current study involve human tissue samples and are therefore not publicly available due to ethical and privacy restrictions. Anonymized data may be made available from the corresponding author upon reasonable request and with appropriate institutional approval.
